# Glycerol Carbonate as an Emulsifier for Light Crude Oil: Synthesis, Characterization, and Stability Analysis

**DOI:** 10.3390/molecules29204937

**Published:** 2024-10-18

**Authors:** Ana Beatriz Morales Cepeda, Elda Elizabeth Villalobos Neri, Luis Alejandro Macclesh del Pino Pérez, Eric Joaquin Gonzalez Pedraza

**Affiliations:** 1 Centro de Investigación en Petroquímica, Tecnológico Nacional de México/Instituto Tecnológico de Cd. Madero, Altamira 89600, Mexico; macclesh_27@yahoo.com (L.A.M.d.P.P.); eric_pedraza1507@hotmail.com (E.J.G.P.); 2 Unidad de Investigación y Tecnología Aplicadas, Facultad de Química, Universidad Nacional Autónoma de México, Vía de la Innovación 410, Autopista MTY-Aeropuerto Km. 10, Parque PIIT, Apodaca 66629, Mexico; elda.neri@hotmail.com

**Keywords:** glycerol carbonate application, biodegradable emulsifier, light crude oil

## Abstract

This study focuses on the synthesis and application of glycerol carbonate (GC) as an emulsifier for light crude oil. GC was synthesized from glycerol and dimethyl carbonate via transesterification, achieving a 90% yield. Characterization through ^1^H-NMR, ^13^C-NMR, and FT-IR confirmed its structure. The emulsification properties of GC were tested by mixing it with light crude oil and water, demonstrating effective emulsification and forming stable emulsions. Stability tests with GC concentrations of 60/40, 70/30, and 80/20 revealed that emulsions remained stable for over 24 h. A particle size analysis indicated that higher GC concentrations produced smaller droplet sizes, enhancing the emulsification efficiency. This study highlights the potential applications of GC in oil spill remediation and enhanced oil recovery, emphasizing its biodegradability and low toxicity as environmental benefits. Overall, GC is presented as an effective and eco-friendly emulsifier for light crude oil, offering stable emulsions and promising industrial applications.

## 1. Introduction

Emulsions are systems composed of two immiscible liquids, where one is dispersed in the form of small droplets, known as the internal phase or droplet phase, while the other is considered the continuous phase. These two phases are usually stabilized by a third component known as an emulsifying agent [1,2]. However, due to their thermodynamically unstable nature, emulsions tend to separate due to various physicochemical mechanisms [3,4,5].

Emulsifiers play a crucial role in the stability of emulsions. The appropriate selection of an emulsifier depends on the nature of the final product [6]. In a water-in-crude oil (*w*/*o*) emulsion, water is dispersed in the form of droplets (internal phase) encapsulated by a matrix of crude oil (continuous phase). In the petroleum industry, the formation of emulsions particularly benefits viscosity reduction, facilitating transport and reducing economic costs [7].

The viscosity of these emulsions depends on several factors, such as the content of oil and water, the temperature, the shear stress, and the amount of solids present in crude oil [8]. Various methods are employed to reduce the viscosity of crude oil and to facilitate its transport through pipelines, such as dilution with alcohols, heating, and the use of surfactants to stabilize the emulsion [9,10].

It is important to note that most currently used surfactants are of petrochemical origin, which pose certain environmental risks and are subjected to environmental regulations [11]. Some long-chain fatty acid esters may be saturated or unsaturated with functional groups, such as hydroxyl groups, epoxides, or glycerol, which are used in many applications as supports in complex formulations [12]. There is significant interest in finding alternatives to ethoxylated emulsifiers because non-ionic emulsifiers are widely used and often released into the environment [13]. Many surfactant solutions include short polyoxyethylene chains, which have been found to be highly toxic and consequently have a significant impact on aquatic life when released into the environment [14,15].

**Figure 1:**
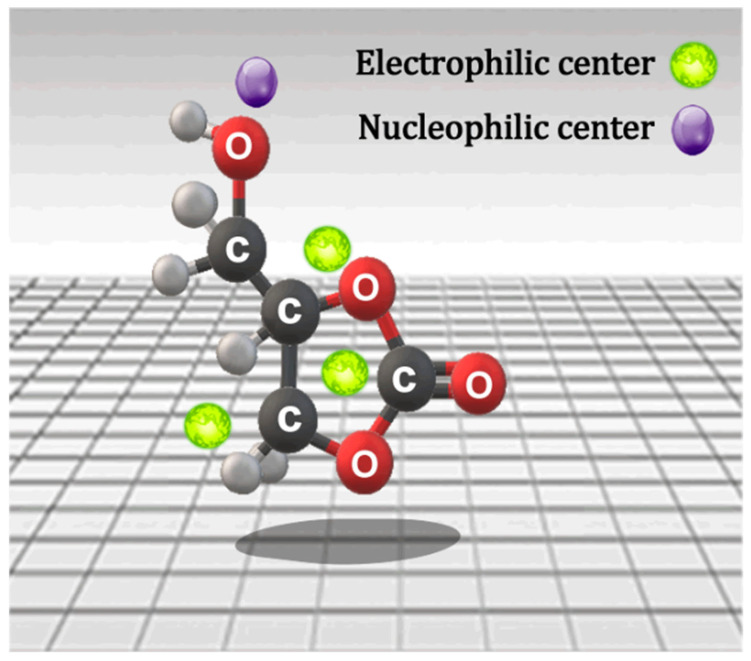
Active centers of glycerol carbonate.

In the search for alternative surfactants, a glycerin derivative known as glycerol carbonate (GC; 4-hydroxymethyl-1,3-dioxolan-2-one) has emerged as a promising candidate. This molecule, which contains hydroxyl groups and a 2-oxo-1,3-dioxolane group, features both an electrophilic center and a nucleophilic center in its structure (see Figure 1), enabling it to react with other functional groups [16,17,18].

The versatility of GC in its applications is due to its structure and physical properties. Notably, it has a high boiling point (110–115 °C at 0.1 mmHg), a high flash point (190 °C), and low volatility (vapor pressure of 8 mmHg at 177 °C). Additionally, it is widely used as an organic solvent in various applications [19,20,21].

GC exhibits both ionization and dissociation capabilities, making it a highly effective solvent. For example, Zhang et al. [22] successfully used it to extract sugar from bagasse, achieving superior results compared with acid pretreatment. GC is also used in the telomerization of butadiene with carbon dioxide to form δ-lactones, which are valuable intermediates in numerous reactions [23].

In addition to its properties as a surfactant, a highlight of glycerol carbonate is that it is a biodegradable molecule, making it an attractive candidate for use in the petroleum industry [24].

The formation of micelles in oil/water mixtures to reduce shear stress is crucial for facilitating the transportation of oil [25]. Although various surfactants and ionic liquids have been used in emulsion formation, many of them pose a risk of degradation into intermediate products that could have a negative environmental impact. Therefore, this study proposes the synthesis of glycerol carbonate from glycerin and its application as a sustainable emulsifier in the interaction with light oil for emulsion formation.

## 2. Results and Discussion

### 2.1. Characterization of GC

**Figure 2:**
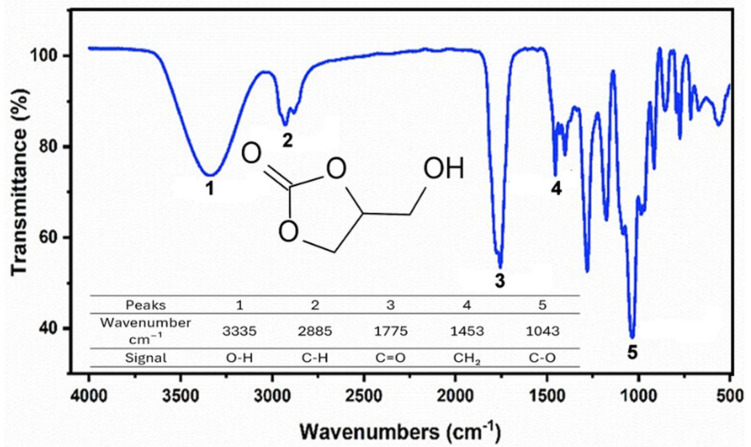
FTIR spectrum 2.2 of glycerol carbonate.

Figure 2 shows the spectrum of the product obtained from the synthesis of GC according to its structure. The presence of an intense band at 1043 cm^−1^ is evident, corresponding to C-O stretching of the glycerol group of the 2-hydroxyethyl chain [26]. Near 1775 cm^−1^, a characteristic band of C=O stretching is present, and at 1453 cm^−1^, a peak attributed to CH_2_ bending is present. Additionally, a band related to C-H stretching appears at 2885 cm^−1^, and at 3335 cm^−1^, stretching of the O-H groups is observed, with bending also present near 1300 cm^−1^ [27,28,29,30].

### 2.2. Chemical Structure Determination of GC

The chemical structure of the GC product molecule was determined using NMR spectroscopy. Kaur et al. [31] demonstrated the influence of the used solvent, as shifts were present in the signals when using D_2_O or DMSO. In the present study, D_2_O was used as the solvent (Sigma-Aldrich, 99% purity, Saint Louis, MO, USA).

**Figure 3:**
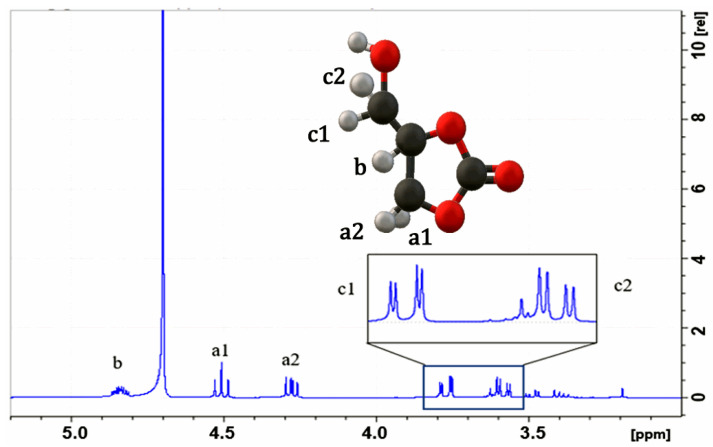
^1^H NMR spectrum of glycerol carbonate; solvent = D_2_O.

Figure 3 presents the proton (^1^H) spectrum, where the signal corresponding to proton a1 is observed in the region of [4.48–4.53 ppm], while that of proton b is found in the region of [4.25–4.3 ppm]. Additionally, protons c1 and c2 are detected in the ranges of [3.75–3.8 ppm] and [3.56–3.64 ppm], respectively. These signals coincide with those reported by Kaur et al. [31]. for glycerol carbonate when analyzed with NMR using D_2_O as the solvent.

**Figure 4:**
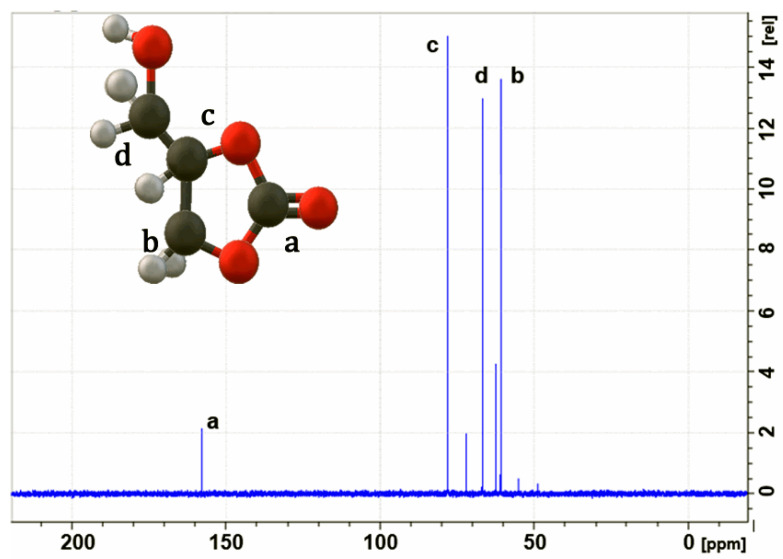
^13^C NMR spectrum of glycerol carbonate; solvent = D_2_O.

Furthermore, Figure 4 shows the 13C spectrum of glycerol carbonate with signals at [159 ppm] (a), [60 ppm] (b), [70 ppm] (c), and [68 ppm] (d), which are similar to those reported by Michele et al. [32]. These results conclusively confirm the structure of glycerol carbonate, demonstrating successful synthesis.

### 2.3. Critical Micelle Concentration (CMC)

**Figure 5:**
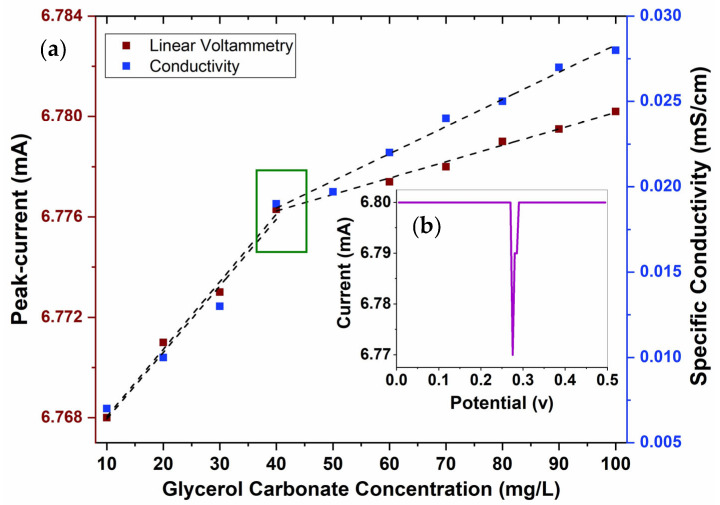
Conductivity results at various concentrations of GC and determination of CMC by linear voltammetry: (**a**) voltammogram at 30 mg/L of GC and (**b**) maximum current values at the various concentrations of GC.

CMC values are typically identified by a sudden shift in a physical property within a narrow concentration range of surfactants. However, variations in the values for the same surfactant can occur, depending on the methods used to analyze the behavior of the surfactant in bulk solution. The results obtained using the conductometric technique are shown in Figure 5. A change in the linear trajectory can be observed when increasing the concentration of GC between 40 and 50 mg/L, resulting in an abrupt change in behavior associated with the CMC. Zdziennicka et al. [33] studied the CMC of various surfactants and similarly observed abrupt changes in their trajectories due to micellization. By extending the regression lines, a crossing point at the value of 44 mg/L was obtained, which represents the CMC for GC. For the pre- and post-micellar regions, regression coefficients of 0.985 and 0.987 were obtained, respectively. This indicates that GC behaves as a typical surfactant with a clear CMC. Figure 5 shows the values found after determining the CMC of GC using the linear sweep voltammetry technique with various concentrations.

### 2.4. Interaction with Light Crude Oil

#### 2.4.1. Preparation of Emulsions and Stability

With the determination of the CMC of GC in water (around 40 mg/L), a value close to this measurement was used (40 mg/L) to form emulsions. A total of six emulsions of 10 mL volume each were prepared, i.e., three without GC and the remaining with GC at a concentration close to its CMC, initially incorporated into the aqueous phase and then mixed with the oil phase under the previously mentioned conditions. Table 1 shows the results obtained for the stability of the emulsion over time, both in the presence and absence of GC. In both cases, the emulsion is more stable in a 70/30 ratio. However, the decrease in emulsion formation time when GC is present, as the interacting forces decrease due to the action of GC, is worth noting [34,35].

#### 2.4.2. Superficial and Interfacial Tension and Surface Tension Reduction Effectiveness (∏cmc)

To corroborate the surface tension of GC, a dropper was filled with water, which is known to have a surface tension of γ = 72 mN/m [31]. Ten drops of water were dropped onto the tray of a balance, and the mass was measured. Subsequently, the same process was carried out with GC, dropping the same number of drops onto the balance tray, and their mass m′ was measured. Tate’s law [36] states that the relationship must satisfy the following conditions:

(1) $$\frac{m}{m\prime }=\frac{\gamma }{\gamma \prime }$$

(2) $$\frac{573}{319}=\frac{72\frac{mN}{m}}{\gamma \prime }=40.52\frac{mN}{m}$$

**Figure 6:**
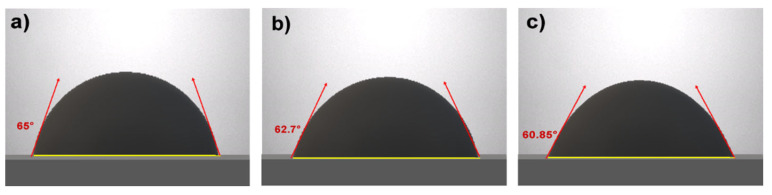
Emulsion sessile drops: (**a**) 60/40, (**b**) 70/30, and (**c**) 80/20.

The process yielded a value of 40.52 mN/m, which is very close to the value reported by S. Mateo [37], confirming the presence of glycerol carbonate. In the case of emulsions, the drop weight method was followed. For the determination of interfacial tension, a drop of crude oil was taken, and its reference contact angle was measured, which was 71°. The contact angles of the emulsions were recorded as shown in Figure 6 being the darker color emulsion drop.

To demonstrate the viability in reducing the surface tension of the emulsions, it was analyzed through the effectiveness in surface tension reduction (∏cmc), which is defined as follows:

(3) $$\prod_{cmc}=\gamma_{0}-\gamma_{cmc}$$

where γ_0_ is the surface tension of distilled water, and γ_cmc_ is the surface tension of the solutions [37,38,39,40]. Table 2 shows the results obtained from the emulsions with and without GC, where a decrease in surface tension is observed in the emulsions containing GC compared with those without it, indicating that GC reduced the forces present for emulsion bonding, thereby lowering the surface tension value.

The data from Table 2 indicate that glycerol carbonate is highly effective in reducing the interfacial tension of crude oil and water emulsions. The 60/40 emulsion shows an interfacial tension of 30.7 mN/m, which is within the common range for light crude oils. However, the 70/30 and 80/20 emulsions demonstrate significant reductions to approximately 17.15 mN/m, which is considerably lower than the typical values for light crude oil without additives, where interfacial tensions range between 30 and 36 mN/m [41]. The notable decrease in interfacial tension observed in the 70/30 and 80/20 emulsions indicates a significant improvement in emulsification, suggesting that these glycerol carbonate proportions are optimal for promoting emulsion stability. Glycerol carbonate likely reduces interfacial tension by adsorbing at the crude oil–water interface, forming a barrier that decreases interfacial energy. This adsorption may be more effective at certain concentrations of glycerol carbonate, explaining the superiority of the 70/30 and 80/20 blends in terms of interfacial tension reduction [42].

**Figure 7:**
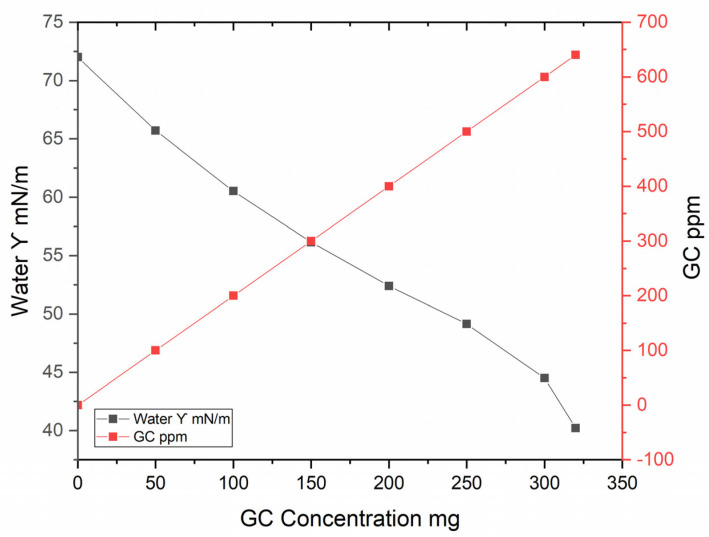
Behavior of surface tension of water vs. GC concentration.

As observed in Figure 7, the surface tension of water decreases progressively as the concentration of glycerol carbonate increases. This behavior indicates that glycerol carbonate acts as a surfactant, reducing the intermolecular forces at the water surface. At a concentration of approximately 300 mg, the surface tension approaches minimum values, suggesting a saturation of the surfactant effect in the solution.

The ∏cmc values indicate that all studied emulsions have significant effectiveness in reducing surface tension. The 70/30 and 80/20 blends show higher ∏cmc values, suggesting that these proportions are optimal for emulsifying crude oil and water using glycerol carbonate. Previous studies have shown that traditional emulsifiers like Tween 80 and Span 80 [43,44] exhibit ∏cmc values in a similar range; however, glycerol carbonate offers the advantages of a lower toxicity and a higher biodegradability. Glycerol carbonate reduces surface tension by forming an interfacial layer between crude oil and water, thereby reducing the energy required to form the emulsion. The effectiveness observed in the 70/30 and 80/20 proportions may be attributed to better distribution and orientation of glycerol carbonate molecules at the interface.

### 2.5. Optical Microscopy

**Figure 8:**
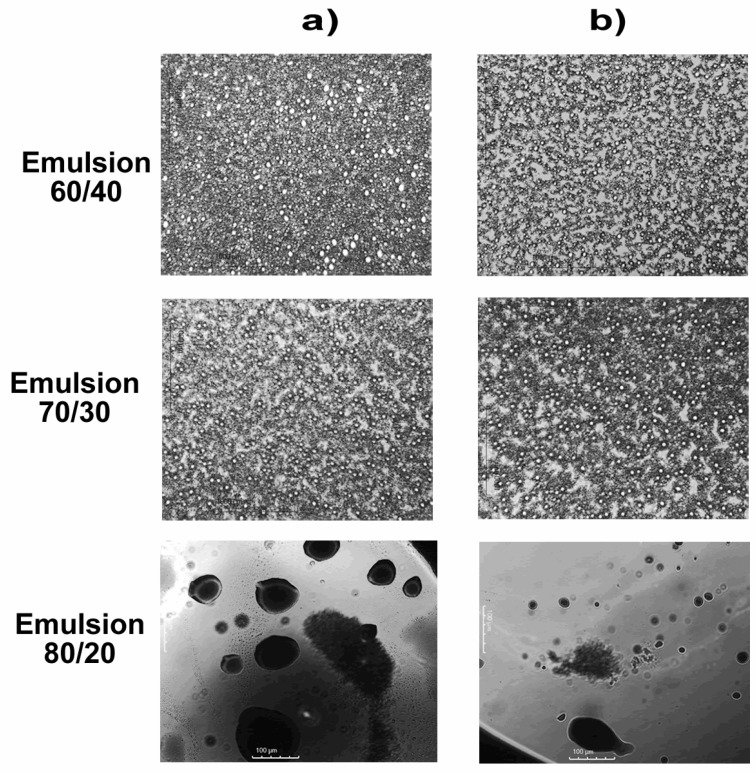
Optical microscopy of emulsions of crude oil/water blends 60/40, 70/30, and 80/20: (**a**) without GC and (**b**) with GC.

Micrographs of the emulsions were taken at a scale of 1000 μm to evaluate the distribution and size of droplets generated by glycerol carbonate acting as an emulsifying agent. The micrographs are presented in Figure 8.

In the micrographs of Figure 8a for 70/30 and 60/40 emulsions, spherical micelle distributions of significantly similar sizes can be observed, which can be attributed to the flocculation process, where small droplets coalesce to form larger droplets. However, this observation may seem contradictory to the results of surface and interfacial tension, which show considerable reductions in these mix ratios. On the other hand, in the micrographs of the 80/20 emulsions (in the section a), an increase in droplet size compared with that of the previous mixes is apparent. This suggests the formation of irregularly shaped micelles with a circular tendency, indicating greater polydispersity in the emulsions [45].

It is crucial to consider that the presence of differently sized micelles and the observation of increased polydispersity in the emulsions do not necessarily contradict the results of surface and interfacial tension. These phenomena can be explained by the complexity of interactions between glycerol carbonate, crude oil, and water (section b). The observed reduction in surface and interfacial tension could be related to glycerol carbonate’s ability to form an effective interfacial layer, thereby reducing the surface energy between crude oil and water. However, the micelles observed in optical microscopy could result from additional interactions, such as the mentioned flocculation, which can influence the rheological and viscoelastic properties of the emulsions.

## 3. Materials and Methods

### 3.1. Synthesis and Characterization of GC

For the synthesis of GC, 80 mL of dimethyl carbonate (DMC, Sigma-Aldrich, 99% purity, Saint Louis, MO, USA) and 12.7 mL of glycerin (Fermont, 99% purity, Monterrey, Mexico) were added to a two-neck flask connected to a reflux condenser and a recirculation system. The mixture was preheated by subjecting it to constant magnetic stirring at a temperature of 75 °C for approximately 10 min. Then, commercial-grade calcium oxide (CaO) was added as a catalyst in a 0.1 molar ratio of catalyst/glycerin.

The CaO was previously dried at 100 °C for 24 h to remove any moisture. The reaction was carried out for 90 min. Upon completion, the catalyst was filtered out, and the reaction products were vacuum-distilled to separate the produced methanol, unreacted DMC, and GC. The molecular structure obtained from the GC synthesis was confirmed via Fourier Transform Infrared Spectroscopy (FTIR) using a Perkin Elmer Spectrum One spectrometer (San Diego, CA, USA) in the range of 400–4000 cm^−1^ with a resolution of 4 cm^−1^ and 12 scans (Shelton, CT, USA), and nuclear magnetic resonance (NMR) using a Bruker Avance III HD 400 MHz NMR spectrometer (Berlin, Germany).

### 3.2. Critical Micelle Concentration (CMC) of GC

The CMC of GC in water was determined using two different techniques: first, via conductometry using a HI2315 conductivity meter from HANNA Instruments (Smithfield, VA, USA), and then, via linear sweep voltammetry (LSV) using a Metrohm 910 PSTAT potentiostat (Herisau, Switzerland), employing a three-electrode film sensor with a 4 mm carbon working electrode, a silver reference electrode, and a carbon auxiliary electrode on a ceramic substrate. Ten different concentrations of aqueous GC solutions in deionized water were tested (10 to 100 mg/L in increments of 10 mg/L). The measurements were performed in triplicate for both techniques, and the average was determined [45,46,47,48,49,50,51,52,53].

### 3.3. Light Crude Oil

The oil was extracted from the Aragón well, located in the Chicontepec region in Central Mexico. This oil, as determined via a Saturates, Aromatics, Resins, and Asphaltenes (SARA) analysis, has an average composition of 25% saturated hydrocarbons, 42% aromatic hydrocarbons, 32% resins, and 0.6% asphaltenes, with a density of 31 API. Additionally, it has a density of 0.878 g/cm^3^ and a viscosity of 18.1 cP [54].

### 3.4. Preparation and Stability of Emulsions

Water-in-oil emulsions were prepared in proportions of 80/20, 70/30, and 60/40 crude oil/water, respectively, by homogenizing the aqueous phase with the oil phase in the presence and absence of a defined concentration of GC under agitation at 4000 rpm for 3 min at an ambient temperature of 28 °C.

To determine the stability of the emulsions, immediately after agitation, 10 mL of the emulsion was poured into glass containers with screw caps (2.3 cm inner diameter, 5 cm height) at room temperature and left to stand. The height of the phase separation interface was recorded to measure the stability of the emulsion over time. The experiment was repeated three times under the same experimental conditions [55,56,57,58].

### 3.5. Optical Microscopy

The emulsions were gently agitated in a glass test tube before analysis to ensure homogeneity. A drop of emulsion was placed on a microscope slide and covered with a cover slip. The microstructures of the emulsions were then observed using a conventional Zeiss AXIO Lab1 optical microscope (New York, NY, USA) connected to the Motic Images Plus 3.0 digital imaging software. Images were taken of each emulsion, and a representative sample was selected for each mixture.

### 3.6. Interfacial and Surface Tension

The surface tension of the emulsions was determined using the drop weight method [59], which involves placing a drop of the substance of interest on a substrate and measuring the contact angle, diameter, and height of the drop. The substrate used was manually polished 304 stainless steel, and the measurement was performed with the Attension^®^ tensiometer (Gothenburg, Sweden). Each measurement was carried out in triplicate, and an average was obtained for each case.

**Figure 9:**
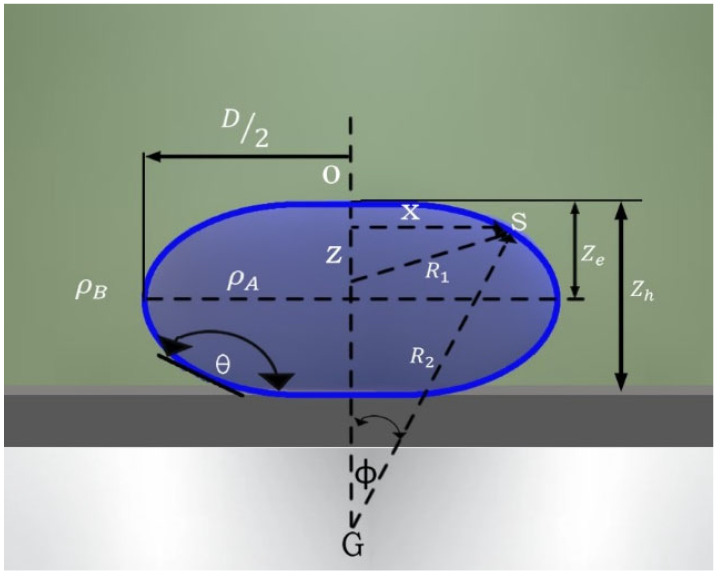
Illustration and coordinates of a sessile drop.

Interfacial tension causes interfaces to behave like membranes and tends to compress the liquid. The shape of an interface in a gravitational field (Figure 9, light blue drop interface and dark blue drop body), depends on the competition between its capillary and gravitational forces and can be estimated using the Bashforth–Adams equation [22]:

(4) $$\gamma \left(\frac{sin\phi }{x}+\frac{1}{R_{1}}\right)=\frac{2\gamma }{b}+∆\rho gz$$

The equation is often expressed in its dimensionless form:

(5) sinϕxb+1R1b=2+∆ρgb2γzb

where γ is the interfacial tension; Δρ is the density difference between the fluids (Δρ = ρA − ρB); R_1_ is the radius of the curvature; x is the rotational radius of point S around the z axis; ϕ represents the angle of vector R_2_ with the symmetry axis; b is the radius of the curvature at its apex; and g is the acceleration due to gravity. If the contact angle is less than 90°, the expression can be described as follows:

(6) $$\gamma =\frac{1}{2}∆\rho gz_{h}^{2}\left[1+0.61\frac{z_{h}}{r}\left(1-\frac{4z_{h}^{2}}{r^{2}}\right)\right]$$

## 4. Conclusions

In this study, the effectiveness of glycerol carbonate (GC) as a sustainable emulsifier for water-in-oil emulsions with light crude oil was investigated. Through various analytical techniques, GC synthesized from glycerin and dimethyl carbonate was characterized, and its chemical structure was confirmed using infrared spectroscopy and nuclear magnetic resonance. GC was found to exhibit a critical micelle concentration (CMC) of approximately 42 mg/L, indicating its ability to form an effective interfacial layer between crude oil and water.

The application of GC in the preparation of emulsions showed promising results in terms of stability and interfacial properties. The emulsions with GC demonstrated a significant reduction in surface and interfacial tension, particularly notable in the 70/30 and 80/20 crude oil/water mix ratios. These optimal GC proportions showed higher effectiveness in reducing surface tension, comparable to traditional emulsifiers like Tween 80 and Span 80, but with the additional advantages of being less toxic and more biodegradable.

Optical microscopy revealed a distribution of emulsified micelles with varied sizes and shapes, influenced by processes such as flocculation. Although some emulsions exhibited higher polydispersity, this did not compromise the overall effectiveness of GC as an emulsifier. Glycerol carbonate emerges as a viable and sustainable alternative for enhancing the stability of crude oil and water emulsions, offering significant benefits in terms of reducing interfacial tension and improving emulsifying properties.

## Figures and Tables

**Table 1:** Results of the time taken for crude oil/water emulsions to stabilize in the presence and absence of GC.

Emulsion	Time (Days)	
	Without GC	With GC
80/20	41	19
	40	21
	41	20
70/30	48	25
	49	24
	52	28
60/40	36	13
	34	11
	37	16

**Table 2:** Results obtained from the interface, surface, and effectiveness of the surface tension of crude oil/water and crude oil/water–GC emulsions.

Emulsion	ƴ = mN/m		Interfacial Tension	∏cmc
	Without GC	GC	mN/m	
80/20	37.38	33.02	30.73	68.698
70/30	36.29	32.43	17.15	68.757
70/30	36.29	32.43	17.15	68.757

## Data Availability

The original contributions presented in the study are included in the article; further inquiries can be directed to the corresponding author.
